# Nanomedicine and the tumor immune microenvironment in cancer immunotherapy: a bibliometric and knowledge-mapping analysis

**DOI:** 10.3389/fonc.2026.1895225

**Published:** 2026-09-04

**Authors:** Yanzhen Mai, Yaqin Wang, Weiwei Li, Jiajia Lu, Xi Luo, Xinli Zhuang, Xiaoran Duan, Ziyi Guo

**Affiliations:** 1 Huizhou Health Sciences Polytechnic, Huizhou, China; 2 National Engineering Laboratory for Internet Medical Systems and Applications, The First Affiliated Hospital of Zhengzhou University, Zhengzhou, Henan, China; 3 Department of Rehabilitation Medicine, The First Affiliated Hospital of Zhengzhou University, Zhengzhou, Henan, China

**Keywords:** bibliometric analysis, cancer immunotherapy, immune regulation, knowledge mapping, nanomedicine, nanotechnology, tumor immune microenvironment

## Abstract

**Background:**

Nanomedicine can improve cancer immunotherapy by enabling tumor-selective delivery and active modulation of the tumor immune microenvironment (TIME), yet the global structure and translational trajectory of this interdisciplinary field remain unclear.

**Methods:**

The Web of Science Core Collection and Scopus were searched from inception to February 1, 2025. After deduplication and screening, 1,110 English-language articles and reviews were included. VOSviewer, CiteSpace, Bibliometrix, and Python were used to analyze publication trends, collaboration networks, journal relationships, co-citation structures, citation bursts, and keyword evolution. Non-negative matrix factorization was applied to citation-burst references to identify latent thematic structures.

**Results:**

Publication output increased sharply after 2022, and studies published during 2022–2025 accounted for 79.9% of the dataset. China led in publication volume and institutional productivity, whereas the United States showed strong citation influence. Soochow University, the Chinese Academy of Sciences, and Sichuan University were the most productive institutions. Three major themes emerged: photodynamic therapy combined with immune checkpoint blockade; tumor-microenvironment remodeling and melanoma immunotherapy; and nanomaterial-mediated immune activation involving cell death, antigen release, and adaptive antitumor responses. Temporal mapping demonstrated a shift from passive nanoparticle delivery toward active immune-regulatory nanoplatforms.

**Conclusion:**

By integrating dual-database bibliometrics, citation-burst analysis, temporal knowledge mapping, and topic modeling, this study clarifies the intellectual structure and evolution of nano-immuno-oncology. Future research should prioritize biologically predictive models, reproducible manufacturing, standardized characterization, long-term safety assessment, biomarker-guided patient selection, and clinically scalable designs.

## 1 Introduction

Cancer represents a major and growing global health burden. According to GLOBOCAN 2022, approximately 20.0 million new cancer cases and 9.7 million cancer-related deaths occurred worldwide in 2022, and the annual number of newly diagnosed cases is projected to exceed 35 million by 2050 (1), Cancer progression shaped not only by uncontrolled cellular proliferation but also by immune evasion and complex interactions within the tumor immune microenvironment (2, 3). The cancer–immunity cycle provides a useful framework for understanding antitumor immune surveillance (4); however, its effective execution is often hindered by the dynamic and heterogeneous nature of the tumor immune microenvironment, which consists of immune cells, stromal components, soluble mediators, and metabolic factors (5–7). In many tumors, insufficient immune cell infiltration, impaired antigen presentation, and the enrichment of immunosuppressive populations, including tumor-associated macrophages, myeloid-derived suppressor cells, and regulatory T cells, collectively promote an immunologically “cold” tumor phenotype and reduce therapeutic responsiveness (8, 9).

Current cancer treatment is inherently multimodal, encompassing surgery, radiotherapy, systemic therapies, and immunotherapy; no single modality is universally optimal, as treatment selection depends on tumor type, stage, molecular characteristics, and patient-related factors. Within this framework, immunotherapy has become an important therapeutic strategy by restoring or enhancing antitumor immune responses. However, durable clinical benefit remains limited to subsets of patients because of insufficient tumor-localized immune activation, limited immune-cell infiltration, immune-related toxicities, and primary or acquired resistance (10–13). These limitations highlight the need for strategies that improve tumor-specific delivery and enable more precise modulation of the tumor immune microenvironment (14, 15).

Nanomedicine has emerged as a promising approach at the interface of oncology, immunology, and materials science. Diverse nanoscale platforms, including liposomal, polymeric, inorganic, biomimetic, extracellular vesicle-based, and metal–organic framework systems, have been explored for the delivery of immune checkpoint inhibitors, tumor antigens, nucleic acids, cytokines, and small-molecule modulators (16–18). In addition to improving tumor-targeted delivery, local drug retention, controlled release, and the stability of labile agents, nanomedicine can facilitate the co-delivery of multiple immunomodulatory agents and regulate key immune processes within the tumor immune microenvironment, including antigen presentation, dendritic cell activation, macrophage repolarization, immune checkpoint signaling, and effector T-cell infiltration (19–21). These advances suggest that nanomedicine is evolving from a conventional delivery tool into an integrated immunomodulatory platform for cancer immunotherapy. Nanomedicine encompasses heterogeneous therapeutic platforms rather than a single intervention; therefore, its clinical effectiveness cannot be expressed as a universal percentage. Efficacy varies according to nanoplatform design, therapeutic payload, tumor type, and clinical endpoint and should be evaluated for specific platforms and indications (22).

Affordability and equitable access are also important considerations for the clinical translation of nano-immunotherapy. Patient financial burden varies considerably across health systems; therefore, manufacturing costs, reimbursement, health-system capacity, and financial protection should be considered when assessing the clinical feasibility and accessibility of advanced nanomedicines (23). Despite rapid advances, research at the intersection of nanomedicine, the tumor immune microenvironment, and cancer immunotherapy remains fragmented, with substantial heterogeneity in experimental models, nanomaterial design, immune targets, and therapeutic strategies (24). Conventional narrative reviews, while informative, are limited in their ability to systematically capture large-scale trends and evolving research patterns in such a multidisciplinary field. Bibliometric and knowledge-mapping approaches provide quantitative and visual tools to analyze publication outputs, collaboration networks, intellectual structures, and emerging themes (25). Therefore, by delineating the knowledge structure and developmental trajectory of this interdisciplinary field, this study aims to identify research hotspots and emerging frontiers, inform future research directions, and facilitate the rational design and clinical translation of nanomedicine-based immunotherapies targeting the tumor immune microenvironment. Nanomedicine and the Tumor Immune Microenvironment in Cancer Immunotherapy: A Bibliometric and Knowledge-Mapping Analysis.

## 2 Methods

### 2.1 Search strategy of literature source

Following preliminary keyword identification and refinement, a systematic literature search was conducted in the Web of Science Core Collection (WoSCC) and Scopus databases on February 1, 2025. The search aimed to identify publications related to nanotechnology, the tumor immune microenvironment, and cancer immunotherapy. The search strategy was adapted according to the syntax and field codes of each database. The following Boolean search strategy was used: (Nano*) AND (“tumor immune microenvironment” OR “immune microenvironment” OR “immunosuppressive microenvironment”) AND (“immunotherapy” OR “cancer immunotherapy” OR “tumor immunotherapy”) The search covered records from database inception to February 1, 2025. Only English-language publications were considered, and no additional restrictions on country or research field were applied.

### 2.2 Data integration, inclusion, and exclusion criteria

**Figure 1:**
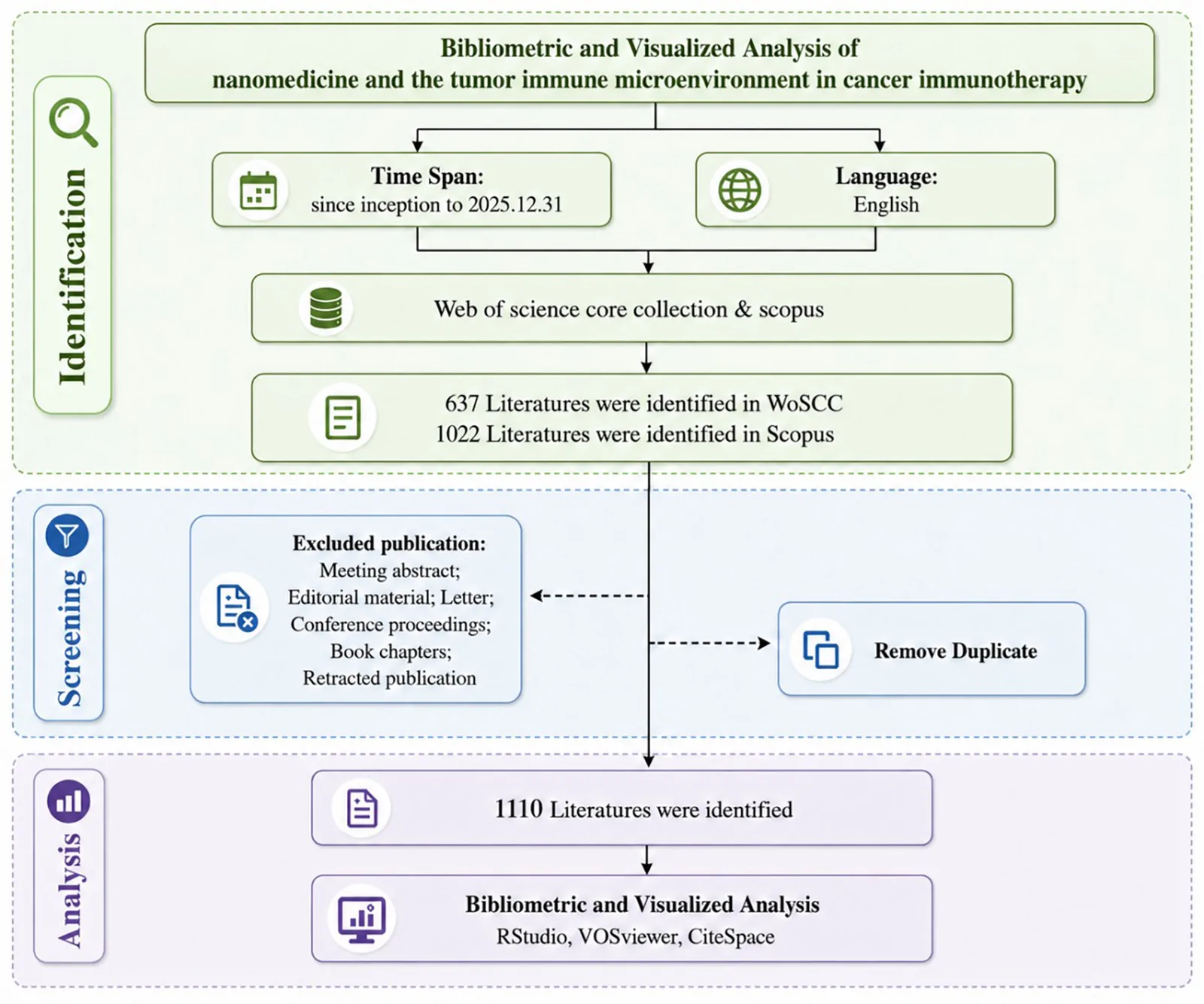
Flowchart of data collection for included studies.

The initial search retrieved 637 records from WoSCC and 1,022 records from Scopus. All retrieved records were exported and imported into Python for data integration, preprocessing, and deduplication. Duplicate records were identified primarily through DOI-based matching. For publications without DOI or with incomplete DOI information, article titles, author names, and publication years were further compared after normalization procedures, including case folding, punctuation removal, and whitespace trimming. Potential duplicate and ambiguous records were manually checked before exclusion. The inclusion criteria were as follows: (1) publications related to nanotechnology, tumor immune microenvironment, and cancer immunotherapy; (2) articles and reviews; and (3) English-language publications. The exclusion criteria included meeting abstracts, editorial materials, letters, conference proceedings, book chapters, retracted publications, duplicate records, and publications irrelevant to the research topic after screening. After deduplication and screening, a final dataset comprising 1,110 publications was retained for subsequent bibliometric and visualization analyses. Bibliometric analyses and visualizations were performed using RStudio, VOSviewer, and CiteSpace. In addition, non-negative matrix factorization (NMF) was employed to identify latent thematic structures among the top references with the strongest citation bursts. The overall workflow of literature retrieval, screening, and analysis is presented in **Figure 1**.

### 2.3 Data preprocessing, bibliometric analysis, and NMF-based topic modeling

Prior to bibliometric analysis, bibliographic records from WoSCC and Scopus were standardized and cleaned to improve data consistency and analytical accuracy. Variations in author names, institutional affiliations, country names, and author keywords were manually checked and merged when necessary. Synonymous keywords and abbreviations were unified to reduce semantic redundancy and improve the reliability of co-occurrence analyses. ORCID iDs, Researcher IDs, and other persistent researcher identifiers were not systematically incorporated into the author-disambiguation procedure. Therefore, although manual standardization was performed to reduce obvious inconsistencies, residual ambiguity related to authors sharing the same surname or initials, abbreviated author names, or changes in institutional affiliation cannot be completely excluded. After preprocessing, the cleaned records were converted into formats compatible with multiple bibliometric analysis platforms. Bibliometric analyses and visualizations were performed using VOSviewer (version 1.6.20), CiteSpace (version 6.2.6 Advanced), RStudio with the Bibliometrix package, and Python. Specifically, VOSviewer was employed to construct and visualize collaboration networks among countries, institutions, and authors, as well as keyword co-occurrence relationships. CiteSpace was used to analyze the knowledge structure and research frontiers of the field through cited reference analysis, citation burst detection, and keyword co-occurrence mapping. Citation burst analysis was applied to identify publications and keywords that experienced rapid increases in scholarly attention over specific time periods, thereby revealing emerging research trends and evolving hotspots. Nodes with high betweenness centrality were considered important bridging points within the knowledge network. The Bibliometrix R-package and its Biblioshiny interface were used for descriptive bibliometric analyses, including publication trends, core journal identification, and temporal evolution of high-frequency keywords.

This study adopts the NMF topic model to identify themes in English literature. First, the original literature data are imported, and the article titles, abstracts, author keywords, and Keywords Plus are combined into a comprehensive text field, so as to retain the thematic information of the literature as completely as possible. Subsequently, the text is preprocessed, including converting all words to lowercase, removing numbers, deleting punctuation marks, and eliminating English stop words, thereby reducing the interference of invalid words in the topic identification results. Based on the preprocessed text, this study applies the TF-IDF method to transform the text into a document-term matrix. TF-IDF can measure both the importance of a term within a single document and its discriminative power across the whole corpus. In this study, both single words and two-word phrases are retained to enhance the identification of professional concepts and compound terms. Python is used as the data processing tool, mainly with libraries such as pandas, matplotlib, and seaborn. Among them, pandas is used to read and organize the topic identification results. Through the above steps, this study establishes a complete processing procedure from raw literature texts to topic identification results. This method can uncover the latent thematic structure of a literature collection in an unsupervised manner and enhance the interpretability of topic model results.

## 3 Results

### 3.1 Annual publication trends and developmental stages

**Figure 2:**
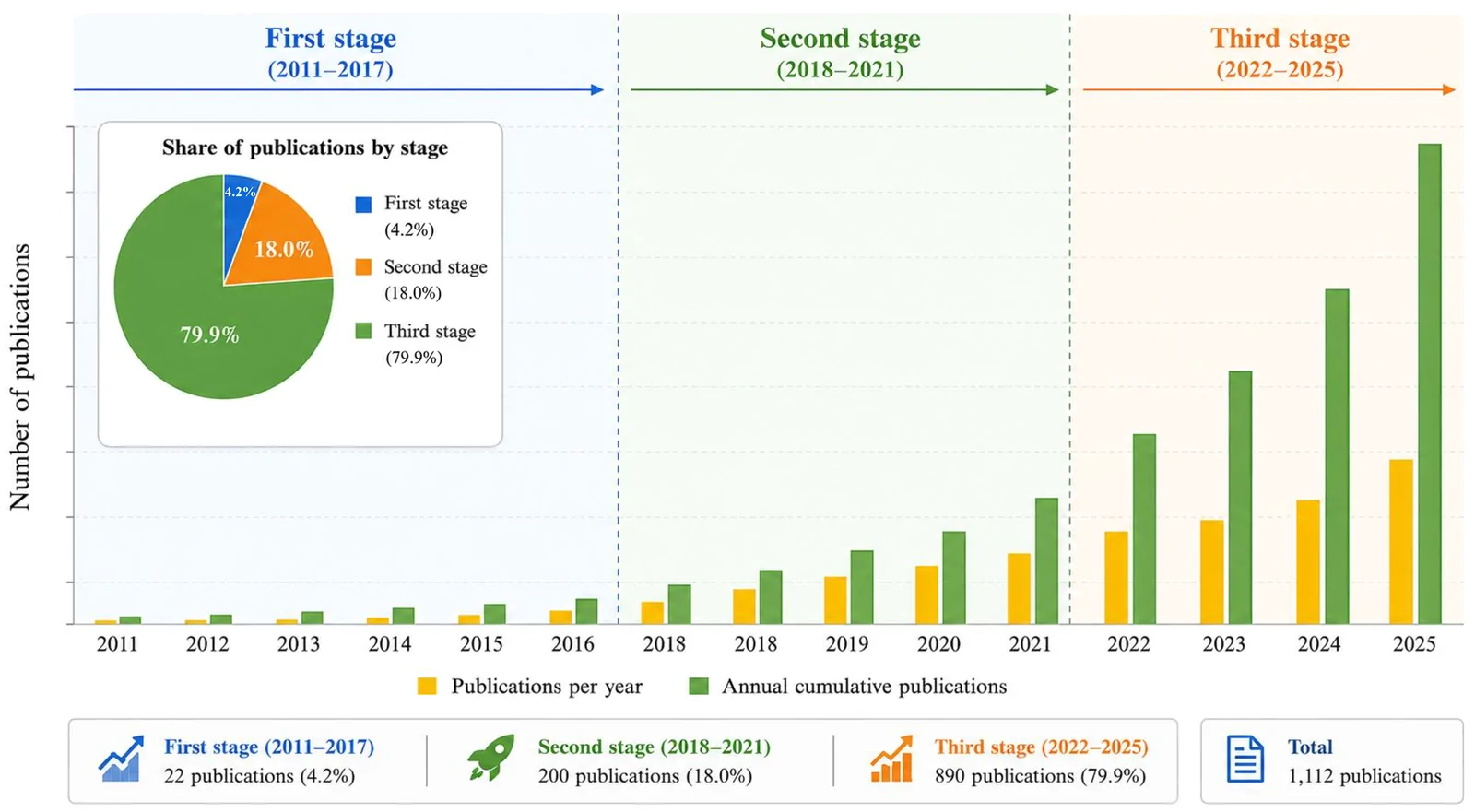
The distribution of publication dates.

To elucidate the developmental trajectory and overarching research momentum of this field, the annual and cumulative publication outputs were quantitatively analyzed. As illustrated in **Figure 2**, the scientific literature concerning nano-enabled tumor immunotherapy exhibits a remarkable, exponential growth pattern from 2011 to 2025. Based on the growth velocity, the temporal evolution of this domain can be distinctly delineated into three developmental stages. The nascent stage, spanning 2011 to 2017 and constituting merely 4.2% of the total output, exhibited low and stable productivity primarily centered on rudimentary nanoparticle design. This was followed by a transitional phase (2018–2021), accounting for 18.0% of publications, which marked a period of rapid acceleration driven by the conceptual integration of nanomedicine with tumor microenvironment modulation. Most notably, the recent explosive stage covering 2022 to 2025 dominates the landscape, representing an overwhelming 79.9% of the literature. This exponential surge confirms that nanoparticle-mediated TME reshaping has matured from an exploratory niche into a globally prioritized frontier in translational oncology. In addition, stage-wise analysis revealed a clear evolution in research priorities from nanoparticle formulation and passive targeting (2011–2017), through tumor microenvironment modulation and nano-enabled immunotherapy (2018–2021), to mechanism-driven immune-engineering strategies (2022–2025). Overall, the field has shifted from materials-centered drug delivery toward mechanism-oriented and translational nano-immuno-oncology.

### 3.2 Country-level collaboration and co-authorship network

**Figure 3:**
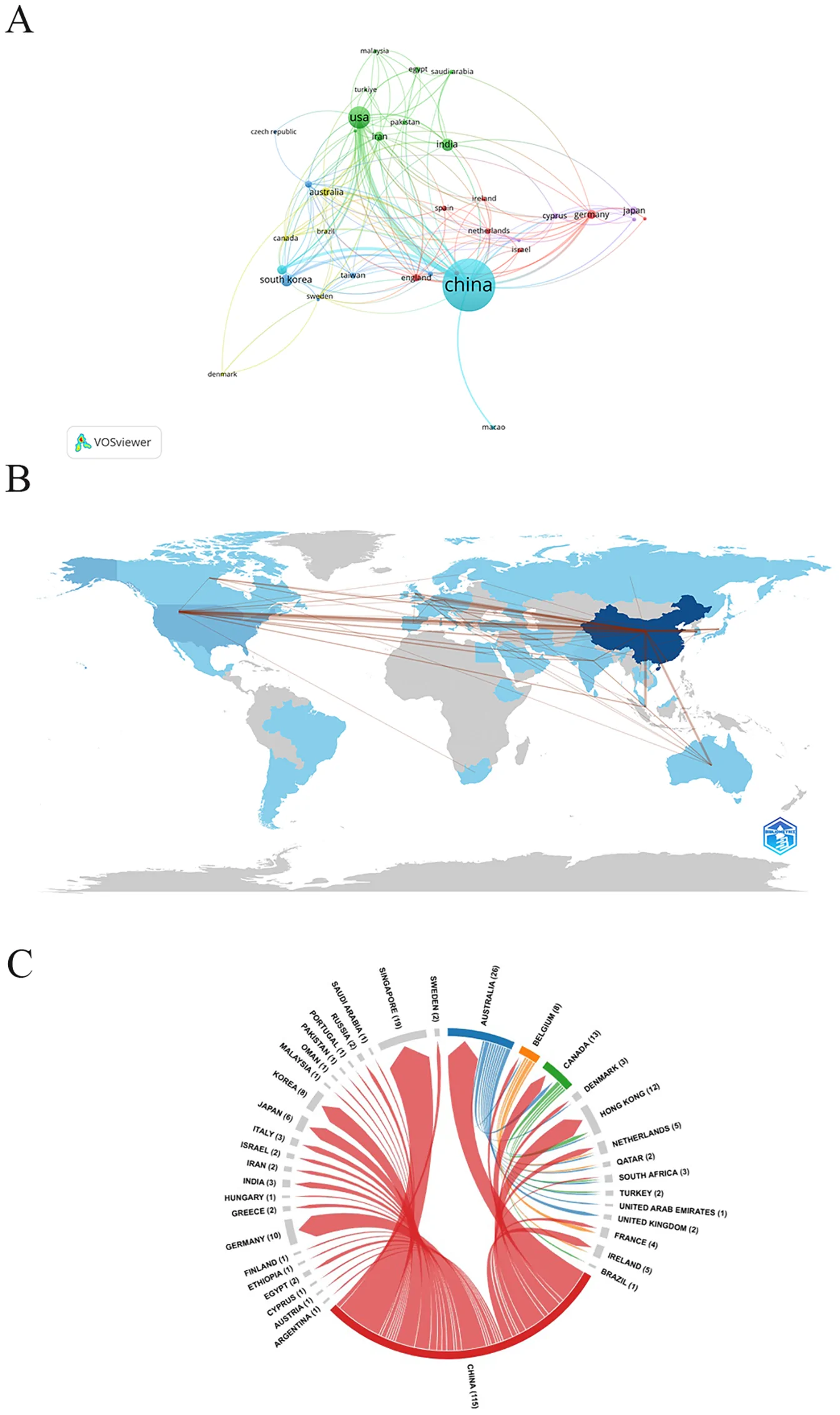
Co-authorship network among countries: **(A)** International research collaboration network. **(B)** global collaboration. **(C)** Chord diagram of international collaborations.

A country-level co-authorship analysis revealed a heterogeneous, polycentric international network in nanotechnology-mediated tumor immune microenvironment and immunotherapy research (**Figure 3A**). China emerged as the dominant hub, leading in both publication volume (830 papers, 68.99% of total) and citations (29,812), underscoring its central role in global collaboration (**Table 1**). The United States (149 publications, 8,273 citations) and several European and Asian countries, including India, South Korea, Singapore, Germany, Australia, Japan, and England, formed secondary clusters with varying connectivity. Notably, Singapore and Japan demonstrated high average citations per paper (73.96 and 64.00, respectively), indicating strong research influence despite smaller output. Geographic mapping (**Figure 3B**) and chord analysis (**Figure 3C**) further highlighted dense collaborative linkages between China, the United States, and select European nations, while other regions exhibited more fragmented interactions. Collectively, these data illustrate a globally active, highly integrated research landscape, with China at the core of international synergy in nano-enabled cancer immunotherapy.

**Table 1:** The top 10 productive countries.

Country	N	Citations	Average citations	%
China	830	29812	35.92	68.99
USA	149	8273	55.52	12.39
India	45	918	20.40	3.74
South Korea	43	1804	41.95	3.57
Iran	27	778	28.81	2.24
Singapore	26	1923	73.96	2.16
Germany	24	1471	61.29	2.00
Australia	22	1193	54.23	1.83
Japan	20	1280	64.00	1.66
England	17	793	46.65	1.41

### 3.3 Core authors and author collaboration networks

**Figure 4:**
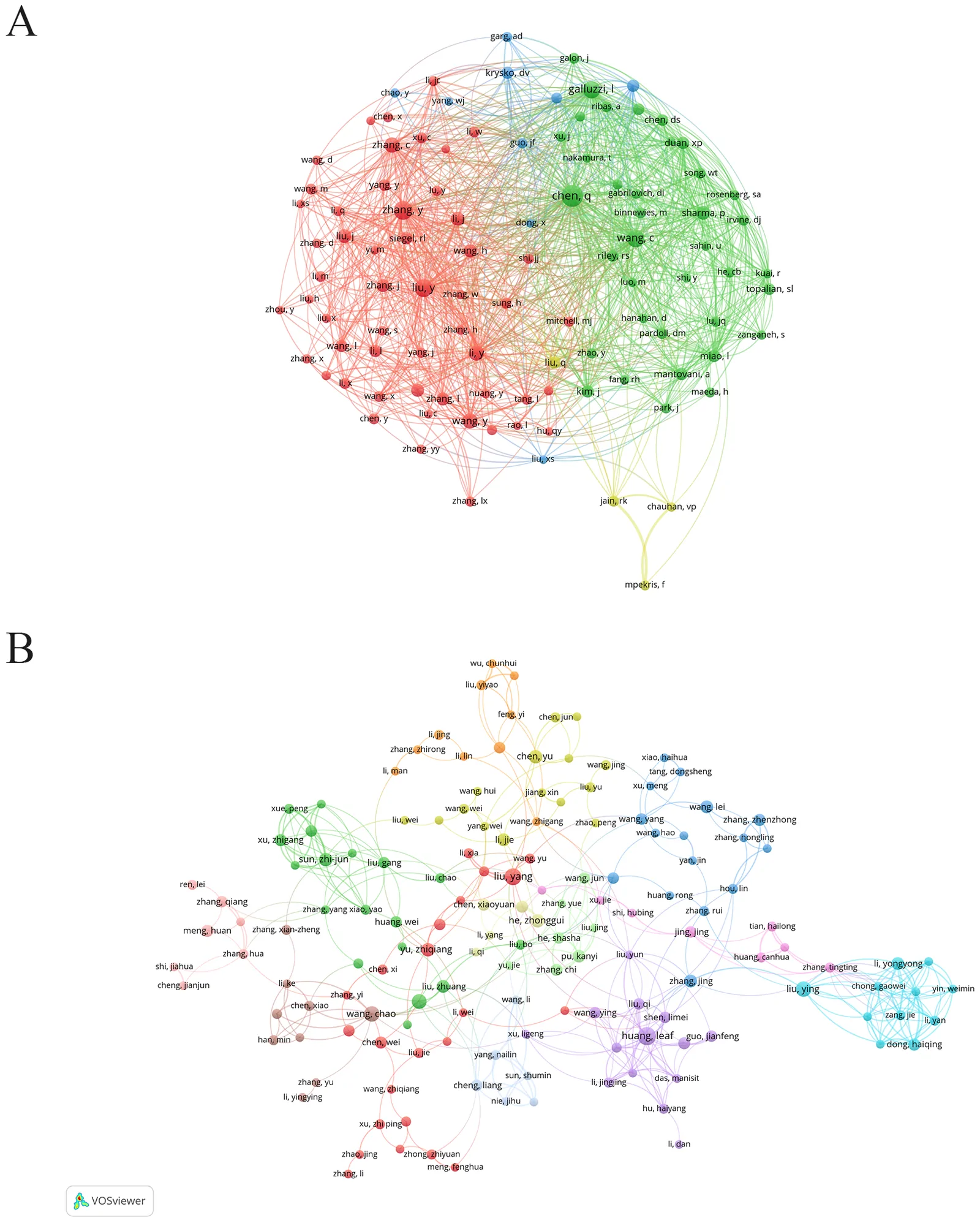
Co-authorship network among core authors: **(A)** co-citation authors **(B)** core authors collaboration.

Author-level network analysis was performed to identify influential contributors and characterize the intellectual and collaborative structure of the field. As shown in **Figure 4A**, the author co-citation network displays a dense and clustered topology, indicating the presence of a relatively cohesive intellectual knowledge base. Two major clusters are particularly evident, representing complementary research domains. One cluster is primarily associated with nanomedicine, drug delivery, and nanoparticle engineering, whereas the other is more closely related to tumor immunology, tumor microenvironment modulation, and cancer immunotherapy. The extensive interconnections between these clusters suggest increasing integration between materials-oriented nanomedicine research and mechanistic immuno-oncology.

Moving from the intellectual knowledge base to direct scientific collaboration, the author co-authorship network (**Figure 4B**) shows a multicentric and modular structure composed of several interconnected research groups. Larger nodes represent authors with higher publication output, while links indicate co-authorship relationships among researchers. Several authors occupy relatively central positions within their respective collaboration modules, and connections between modules indicate the presence of cross-group collaboration. Compared with the more integrated co-citation network, however, the co-authorship structure remains partly modular, suggesting that broader collaboration across research groups may still be strengthened. Taken together, the co-citation and co-authorship networks indicate that nano-immuno-oncology has developed an increasingly interdisciplinary knowledge base supported by multiple active collaborative communities.

### 3.4 Leading institutions

Institutional analysis identified the top ten contributing institutions in this research field (**Table 2**). Soochow University (China) ranked first with 44 publications, followed by the Chinese Academy of Sciences (China, n = 40) and Sichuan University (China, n = 37). Notably, all of the top ten institutions were located in China, reflecting the country’s dominant role and strong research capacity in this area. Among these institutions, Shanghai Jiao Tong University and Wuhan University demonstrated particularly high academic influence, with average citation counts of 43.58 and 58.45 per publication, respectively, while Soochow University achieved the highest total citations (3,479). Collectively, the top ten institutions produced 313 publications and accumulated 14,912 citations, indicating both high productivity and substantial scientific impact. The concentration of leading institutions within China also suggests the existence of strong domestic research networks and collaborative platforms that have contributed significantly to the advancement of this field.

**Table 2:** Top 10 institutions of high contribution.

Institution	Country	Documents	Total citations	Average citations
Soochow University	China	44	3479	79.07
Chinese Academy of Sciences	China	40	2444	61.1
Sichuan University	China	37	1504	40.65
Zhejiang University	China	34	1363	40.09
Fudan University	China	31	1109	35.77
Shanghai Jiao Tong University	China	31	1351	43.58
Ministry of Education of China	China	27	942	34.89
Zhengzhou University	China	24	716	29.83
Huazhong University of Science and Technology	China	23	718	31.22
Wuhan University	China	22	1286	58.45

### 3.5 Journal relationship analysis

**Figure 5:**
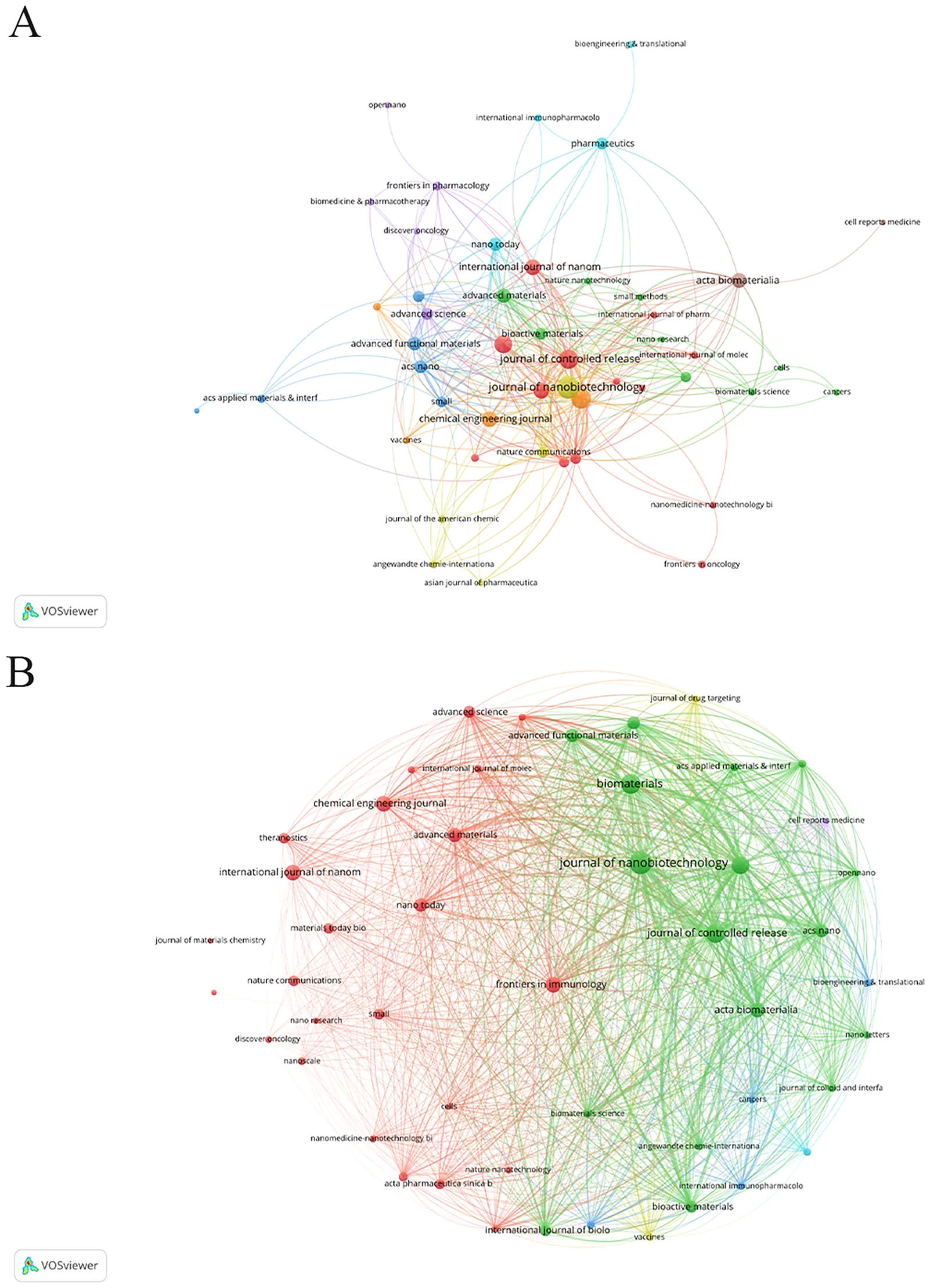
Journal relationship analysis: **(A)** journal citation analysis; **(B)** journal coupling analysis.

Journal-level network analyses were performed to characterize the disciplinary landscape and knowledge structure of the field. As shown in **Figure 5A**, the journal citation network displays a densely interconnected structure, with several journals occupying relatively central positions and linking research across nanotechnology, oncology, immunology, pharmacology, and biomaterials science. These journals serve as important channels for knowledge exchange among the major disciplinary components of nano-immuno-oncology. Complementing the citation analysis, the bibliographic coupling network (**Figure 5B**) groups journals according to similarities in their cited references and therefore reflects shared intellectual foundations and research orientations. Two major and closely connected domains are evident. One is primarily associated with materials science, including nanoparticle design, biomaterials, and drug-delivery engineering, whereas the other is more closely related to translational oncology and immunology, particularly tumor microenvironment regulation and immune checkpoint therapy. Connections between these domains further indicate increasing interdisciplinary integration between materials-oriented nanomedicine and mechanistic immuno-oncology.

**Table 3** summarizes the 10 most productive journals in the dataset. Journal of Nanobiotechnology ranked first with 67 publications and 1,987 total citations (29.66 citations per article), followed by Biomaterials with 54 publications and 2,247 citations (41.61 citations per article) and Journal of Controlled Release with 50 publications and 858 citations (17.16 citations per article). Other major publication sources included Advanced Healthcare Materials, Chemical Engineering Journal, Frontiers in Immunology, International Journal of Nanomedicine, Acta Biomaterialia, Advanced Materials, and Nano Today. Among these journals, Chemical Engineering Journal and Nano Today showed comparatively high average citation counts of 83.33 and 57.52 citations per article, respectively. Collectively, these journals span materials science, biomaterials, nanoscience, pharmacology, oncology, and immunology, highlighting the multidisciplinary nature of nano-enabled cancer immunotherapy research. The citation and bibliographic-coupling patterns further suggest increasing convergence between nanomaterial and drug-delivery research and mechanistic and translational immuno-oncology.

**Table 3:** Top 10 citing sources.

Journal	N	Total citations	Average citations	JCR2025	Discipline
journal of nanobiotechnology	67	1987	29.66	Q1	Biotechnology & Applied Microbiology
biomaterials	54	2247	41.61	Q1	Materials Science, Biomaterials
journal of controlled release	50	858	17.16	Q1	Pharmacology & Pharmacy
advanced healthcare materials	42	967	23.02	Q1	Materials Science, Biomaterials
chemical engineering journal	33	2750	83.33	Q1	Engineering, Chemical
frontiers in immunology	33	400	12.12	Q2	Immunology
international journal of nanomedicine	32	668	20.88	Q2	Nanoscience & Nanotechnology
acta biomaterialia	29	1565	53.97	Q1	Materials Science, Biomaterials
advanced materials	26	871	33.50	Q1	Materials Science, Multidisciplinary
nano today	25	1438	57.52	Q1	Nanoscience & Nanotechnology

### 3.6 Citation burst analysis of foundational literature

**Figure 6:**
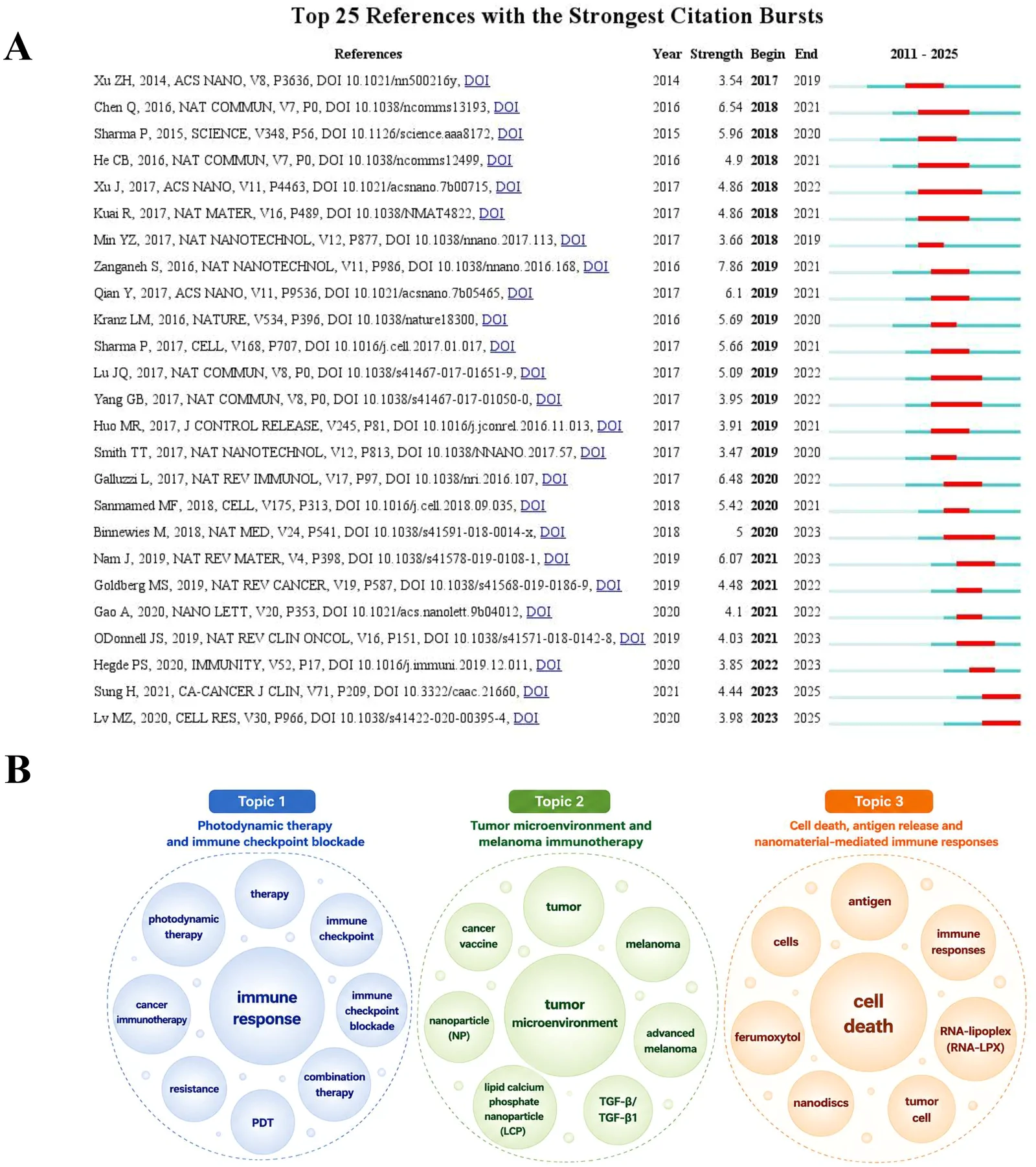
**(A)** Citation burst references and **(B)** NMF-derived thematic structure of research hotspots.

A citation burst analysis was deployed to identify seminal publications that experienced intense, time-specific surges in scholarly recognition, thereby delineating the dynamic evolution of the field’s knowledge base. As illustrated in **Figure 6A**, the top 25 references exhibiting the strongest citation bursts are heavily concentrated within the past decade. These publications, many of which were published in multidisciplinary and specialized journals, help illustrate the evolution of major research themes and methodological developments in this domain. Taken together, the thematic orientation of these high-burst references shows a clear convergence around several emerging research directions, particularly nanoparticle engineering, tumor microenvironment modulation, and the advancement of cancer immunotherapy. The reference with the highest burst strength, Zanganeh et al., further highlights the relevance of mechanistic studies linking nanotechnology with tumor immunology. In addition, the continued presence of burst references during 2023–2025, including Sung et al. (2021) and Lv et al. (2020), suggests that nano-enabled regulation of the tumor immune microenvironment has remained an active and evolving research focus in recent years.

To further characterize the latent thematic structure embedded within these highly cited burst references, we subsequently performed a matrix decomposition analysis (MDA) based on the top 25 references with the strongest citation bursts. The resulting topic structure is visualized in **Figure 6B** through an NMF Topic–Keyword Bubble Map. Three representative thematic clusters were identified. Topic 1, entitled Photodynamic therapy and immune checkpoint blockade, primarily focuses on photodynamic therapy (PDT), immune checkpoint blockade, cancer immunotherapy, combination therapy, and therapeutic resistance. This topic reflects the growing emphasis on synergistic therapeutic strategies designed to enhance antitumor immune responses and overcome immunotherapy resistance. Topic 2, labeled Tumor microenvironment and melanoma immunotherapy, centers on tumor microenvironment regulation, melanoma immunotherapy, nanoparticle-based vaccine delivery systems, and TGF-β-related immunomodulation. Representative keywords such as tumor microenvironment, melanoma, nanoparticle, and lipid calcium phosphate nanoparticle indicate the increasing importance of nanoplatform-enabled remodeling of the immunosuppressive tumor microenvironment. Topic 3, designated Cell death, antigen release and nanomaterial-mediated immune responses, highlights mechanisms associated with cell death, antigen presentation, RNA-lipoplex, nanodiscs, ferumoxytol, and antitumor immune responses. This topic suggests a growing research interest in nano-enabled immune activation mechanisms and antigen delivery platforms that facilitate adaptive antitumor immunity.

### 3.7 Thematic landscape and intellectual evolution

**Figure 7:**
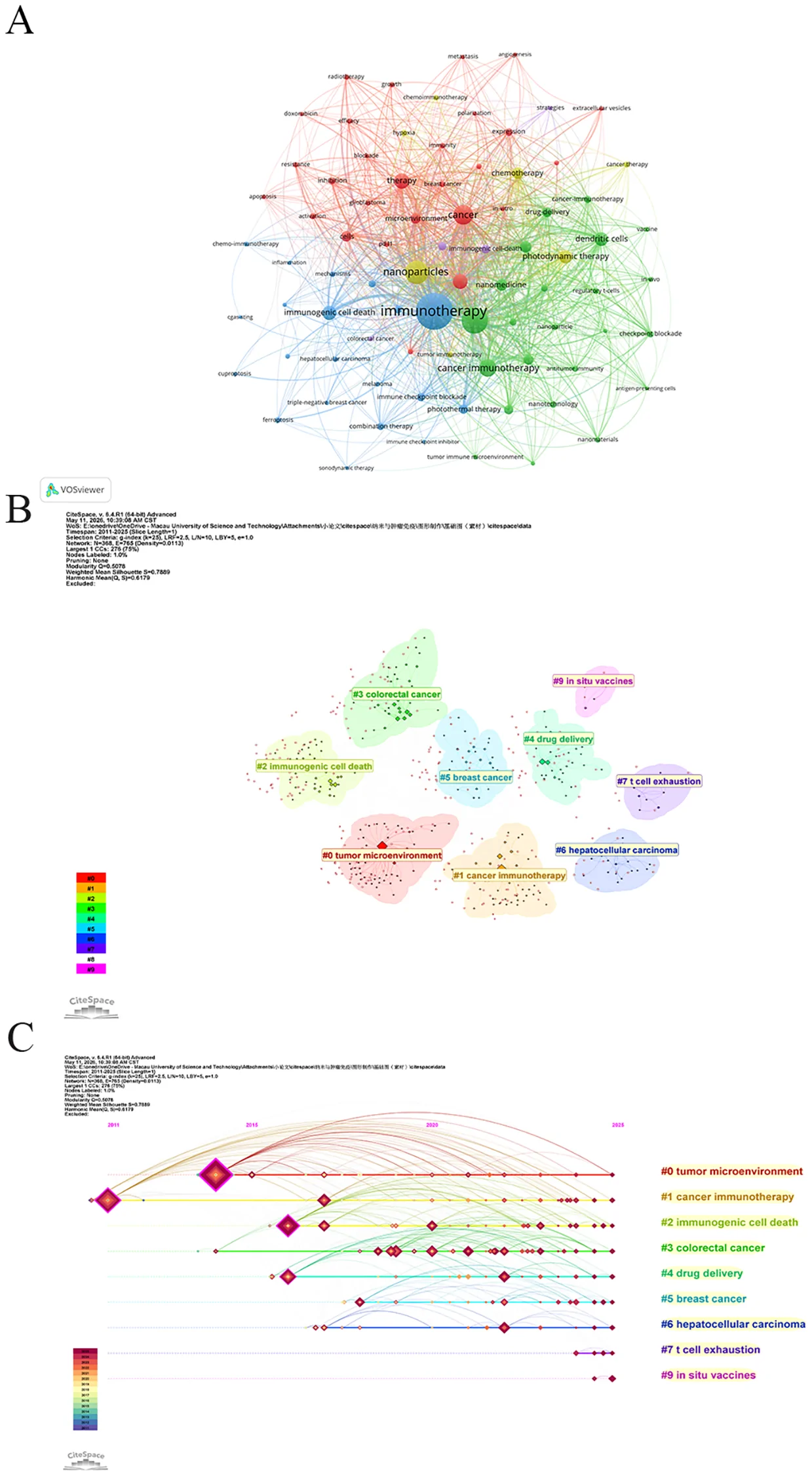
Research hotspots and thematic evolution in nanotechnology-based tumor immune microenvironment and immunotherapy research. **(A)** Keyword co-occurrence network. **(B)** Co-citation cluster map. **(C)** Timeline visualization of co-citation clusters.

To comprehensively map the thematic landscape and intellectual evolution of the field, keyword co-occurrence and reference co-citation analyses were integrated. The keyword network (**Figure 7A**) exhibits a highly interconnected topology anchored by core hubs: “nanoparticles,” “immunotherapy,” and “tumor microenvironment”, verifying that nanoparticle-enabled immunomodulation constitutes the central pillar of current research. This thematic convergence is structurally substantiated by the co-citation cluster map (**Figure 7B**), which delineates the underlying theoretical domains bridging biomaterials and advanced immunology, notably including #0 tumor microenvironment, #2 immunogenic cell death, and #4 drug delivery, alongside specialized frontier applications such as #7 t cell exhaustion and #9 *in situ* vaccines. Furthermore, the timeline visualization (**Figure 7C**) explicitly tracks the chronological trajectory of these research paradigms. While early theoretical foundations were predominantly restricted to classical formulation science and passive drug accumulation, a profound temporal shift reveals that recent activity is heavily concentrated in mechanistic, immune-centric clusters. Collectively, these multilayered analyses demonstrate a decisive conceptual transition: the field has shifted from utilizing nanomaterials merely as “inert delivery vehicles” to engineering them as “dynamic immuno-regulatory platforms” that actively reshape immunosuppressive niches, reverse T-cell exhaustion, and potentiate robust systemic anti-tumor immunity.

## 4 Discussion

### 4.1 Rapid growth reflects the increasing convergence of nanotechnology and tumor immunology

A temporal analysis of publication output reveals a distinct three-stage trajectory in the intersection of nanomedicine, the tumor immune microenvironment, and cancer immunotherapy. Prior to 2017, the field exhibited modest publication activity, reflecting the exploratory nature of early oncological nanotechnology and a nascent understanding of intratumoral immune regulation. Consequently, early research predominantly centered on nanoparticle engineering, drug delivery optimization, and passive targeting strategies. The transitional phase (2018–2021) closely paralleled breakthrough advancements in cancer immunotherapy, notably the widespread clinical success of immune checkpoint inhibitors. As the critical role of the tumor immune microenvironment in therapeutic resistance and disease progression became more evident, research efforts rapidly pivoted towards nano-enabled immunomodulatory approaches. Furthermore, the exponential surge in publications post-2022 signifies an era of profound interdisciplinary synergy, fueled by the convergence of nanotechnology, tumor immunology, biomaterials science, and translational oncology.

From an immunological standpoint, this soaring trajectory underscores a paradigm shift in cancer therapeutics. Transcending their traditional roles as mere passive delivery vehicles, modern nanoplatforms are increasingly engineered as proactive immunoregulatory systems (26). They are now tailored to modulate antigen presentation, reverse localized immunosuppression, facilitate immune-cell infiltration, and amplify systemic antitumor responses (27, 28). Ultimately, this conceptual evolution seamlessly aligns with the modern paradigm of the tumor immune microenvironment as a highly dynamic, therapeutically targetable ecosystem.

### 4.2 Global research landscape reveals dominant contributions from China and an increasingly interconnected collaboration network

Country-level analysis demonstrated substantial heterogeneity in publication productivity, citation impact, and collaborative intensity. China has emerged as the predominant contributor in terms of overall output, institutional productivity, and network centrality, reflecting sustained national investment in nanotechnology, biomaterials, and translational cancer research. In contrast, the United States exhibited a comparatively higher average citation impact, suggesting a profound influence in conceptual innovation and high-impact translational studies. Furthermore, several countries—including Singapore, Germany, Japan, and Australia—demonstrated robust citation rates despite relatively smaller publication volumes, indicating exceptional academic quality and international visibility. These complementary national roles underscore varied research emphases across the globe, ranging from extensive empirical investigations to mechanistic innovation and early-stage immunotherapy development.

The collaboration network further reveals that this field has matured into a globally interconnected research ecosystem. China and the United States serve as primary collaborative hubs linking Asia, North America, and Europe. However, collaboration intensity remains uneven, with certain emerging, research-active countries exhibiting relatively fragmented participation. From the perspective of translational immunotherapy, enhancing international collaboration could further support the harmonization of experimental standards, improve reproducibility, and potentially accelerate the clinical translation of nano-enabled immunotherapeutic strategies.

### 4.3 Structured author and institutional networks reflect increasing interdisciplinary integration

Analysis of core authors and institutions revealed a relatively mature yet modular research community. The co-citation network demonstrated a highly cohesive intellectual structure supported by several influential research groups, while the co-authorship network exhibited multiple interconnected collaborative clusters. Unlike highly fragmented early-stage research areas, the observed network topology suggests that nano-immuno-oncology has developed into a comparatively stable and integrated interdisciplinary field. The author clusters exhibited clear thematic specialization, primarily centered on nanoparticle engineering, tumor microenvironment remodeling, immune checkpoint modulation, photodynamic therapy, and biomimetic delivery systems. This thematic organization perfectly reflects the organic integration of materials science with mechanistic tumor immunology. At the institutional level, China led the research landscape, with all of the top ten most productive institutions located within the country. Institutions such as Soochow University, the Chinese Academy of Sciences, and Shanghai Jiao Tong University demonstrated both high productivity and strong citation influence, highlighting China’s emergence as a global epicenter for nano-immunotherapy research. However, despite robust domestic collaboration networks, broader cross-continental institutional integration remains relatively limited. Given the multifaceted complexity of tumor immunology and the hurdles of nanomedicine translation, future breakthroughs will likely depend on dismantling these modular boundaries, fostering deeper collaborative synergy among oncologists, immunologists, materials scientists, and clinical researchers globally.

### 4.4 Intellectual foundations emphasize tumor microenvironment modulation and nano-enabled immunotherapy strategies

Highly cited references and citation burst analyses provided important insights into the intellectual foundations of the field. Early influential studies primarily focused on nanoparticle-mediated drug delivery, biomaterial engineering, and strategies to improve tumor-targeted accumulation. These foundational studies established the methodological basis for subsequent integration with cancer immunotherapy. Over time, however, the thematic orientation of highly cited literature shifted toward immune-centered therapeutic strategies. Citation burst references increasingly emphasized tumor microenvironment remodeling, immune checkpoint blockade, immunogenic cell death, and synergistic combination therapies. This evolution reflects a major conceptual transition from conventional nanomedicine toward dynamic immunomodulatory systems capable of actively reshaping antitumor immune responses.

Importantly, many of the most influential references focused not only on improving delivery efficiency but also on enhancing mechanistic immune activation. The growing prominence of studies involving photodynamic therapy, RNA-lipoplex systems, nanodiscs, and exosome-mediated immunoregulation suggests that precision immune activation and antigen presentation have become central research priorities. This transition demonstrates that nano-immunotherapy research is increasingly driven by mechanistic immunological objectives rather than formulation optimization alone.

### 4.5 Hotspots and development trends

By integrating keyword co-occurrence analysis, co-citation clustering, citation burst detection, and NMF topic modeling, the major research hotspots and emerging trends in nanomedicine-mediated tumor immune microenvironment research can be summarized into three interconnected themes: (1) nano-enabled remodeling of the immunosuppressive tumor microenvironment, (2) combination immunotherapeutic strategies and immune activation mechanisms, and (3) translational challenges and future clinical perspectives.

#### 4.5.1 Nano-enabled remodeling of the tumor immune microenvironment

One of the most prominent research hotspots identified in this study is the use of nanotechnology to regulate the immunosuppressive tumor microenvironment. Keyword analyses revealed increasing attention to immune-related mechanisms such as macrophage polarization, T-cell exhaustion, cytokine modulation, and immunogenic cell death. These findings reflect the growing recognition that immunotherapy requires not only activation of immune effector cells but also reversal of tumor-induced immunosuppression. Nanotechnology offers several unique advantages for tumor immune microenvironment modulation, including targeted delivery, controlled release, co-delivery of multiple agents, and enhanced intratumoral retention. Nanoplatforms have been widely explored to deliver immune checkpoint inhibitors, cytokines, nucleic acids, and tumor antigens directly into tumor tissues, thereby enhancing local immune activation while minimizing systemic toxicity (29–32). Furthermore, certain nanomaterials may themselves exert intrinsic immunomodulatory effects by influencing macrophage polarization, dendritic-cell maturation, oxidative stress, and inflammatory signaling pathways (33, 34). The increasing integration of nanotechnology with tumor immunology highlights a conceptual transition from passive drug delivery toward active immune engineering strategies designed to reshape the tumor ecosystem.

#### 4.5.2 Combination immunotherapy and nano-enabled immune activation strategies

Another defining hotspot centers on synergistic combination therapies that integrate nanotechnology with modalities such as photodynamic therapy (PDT), immune checkpoint blockade (ICB), and advanced antigen-delivery systems. NMF topic modeling prominently highlighted the PDT-ICB combination cluster, reflecting intense interest in strategies that concurrently induce immunogenic tumor cell death and amplify adaptive immune responses. The prominent emergence of keywords like “cold tumors,” “immune activation,” and “*in situ* vaccination” signals a concerted effort to overcome immunotherapeutic resistance by transforming immunologically inert (“cold”) tumors into immune-responsive (“hot”) phenotypes. In this context, nano-enabled antigen-delivery systems, RNA-lipoplexes, and biomimetic nanocarriers have garnered substantial traction for their ability to optimize antigen presentation and elicit potent systemic antitumor immunity (35–38). Concurrently, extracellular vesicles and biomimetic nanoparticles are emerging as cutting-edge precision immunotherapy platforms (37–41). Their intrinsically low immunogenicity, high biocompatibility, and natural intercellular communication capabilities signify a progressive shift toward highly integrated immune-engineering systems capable of orchestrating multi-step, durable antitumor responses (42, 43).

#### 4.5.3 Translational challenges and future perspectives of nano-immunotherapy

Despite substantial progress, important translational challenges remain. Citation burst and keyword trend analyses revealed persistent attention to issues related to biosafety, delivery efficiency, clinical applicability, and therapeutic reproducibility. Several physiological barriers—including abnormal tumor vasculature, elevated interstitial pressure, heterogeneous immune infiltration, and off-target accumulation—continue to limit nanoparticle delivery efficiency *in vivo* (44–46). while the enhanced permeability and retention effect is highly variable across tumors and patients (47, 48). Moreover, conventional animal models may not adequately reproduce human tumor biology or immune responses, contributing to discrepancies between preclinical efficacy and clinical outcomes (45, 47). Future studies should therefore emphasize quantitative pharmacokinetic and biodistribution analyses, human-relevant experimental models, and biomarker-guided patient stratification to improve the prediction of therapeutic response. Additional barriers include manufacturing complexity, batch-to-batch variability, long-term biosafety, high production costs, and uncertain regulatory pathways. Multifunctional nanoplatforms may provide therapeutic advantages but often complicate scale-up, quality control, and good manufacturing practice implementation. Future development should therefore prioritize simplified and scalable designs, well-defined critical quality attributes, standardized safety assessment, and early regulatory engagement (23, 49). Overall, successful clinical translation will require a better balance between technological sophistication and practical considerations of reproducibility, manufacturability, safety, and accessibility.

## 5 Limitations

This study has several limitations that should be acknowledged. First, only English-language publications indexed in the Web of Science Core Collection and Scopus databases were included, which may have excluded relevant studies indexed elsewhere or published in other languages. In addition, because ORCID iDs and other persistent researcher identifiers were not systematically incorporated into author disambiguation, residual ambiguity may remain for authors with identical or abbreviated names or for researchers who changed institutional affiliations. Second, bibliometric analyses are inherently influenced by citation lag, database coverage, and indexing algorithms, potentially resulting in underrepresentation of newly emerging research topics. Third, citation-based indicators primarily reflect academic influence rather than clinical efficacy or translational success. Finally, although thematic clustering and visualization analyses provide valuable insights into research evolution, they may oversimplify the complex interdisciplinary interactions underlying nano-immunotherapy research.

## 6 Conclusion

This bibliometric and knowledge-mapping analysis provides a comprehensive overview of research at the intersection of nanomedicine, tumor immune microenvironment modulation, and cancer immunotherapy. A key contribution of this study is the integration of dual-database bibliometric analysis, citation-burst detection, temporal co-citation mapping, and non-negative matrix factorization (NMF) to characterize the intellectual structure and thematic evolution of nano-immuno-oncology. The findings reveal rapid publication growth, increasing interdisciplinary integration, and a conceptual shift from conventional nanoparticle-based drug delivery toward active immune-regulatory nanoplatforms. Three major thematic structures were identified: photodynamic therapy combined with immune checkpoint blockade; tumor microenvironment remodeling and melanoma immunotherapy; and nanomaterial-mediated immune activation involving cell death, antigen release, and adaptive antitumor responses. China and the United States emerged as leading and complementary contributors in terms of research output, citation impact, and international collaboration. Current research hotspots center on tumor microenvironment remodeling, immune activation, and combination immunotherapy. Despite substantial progress, important translational challenges remain. Future advances will require closer integration of nanotechnology, immunology, biomaterials science, and precision oncology to promote the clinical translation of safe, effective, and personalized nano-immunotherapies.

## Data Availability

The original contributions presented in the study are included in the article/supplementary material. Further inquiries can be directed to the corresponding authors.
